# Immunomodulation as a Therapy for *Aspergillus* Infection: Current Status and Future Perspectives

**DOI:** 10.3390/jof4040137

**Published:** 2018-12-14

**Authors:** Chris D. Lauruschkat, Hermann Einsele, Juergen Loeffler

**Affiliations:** Department of Internal Medicine II, University Hospital Wuerzburg, WÜ4i, Building C11, 97080 Wuerzburg, Germany; Lauruschka_c@ukw.de (C.D.L.); Einsele_h@ukw.de (H.E.)

**Keywords:** Immunotherapy, invasive aspergillosis, *Aspergillus fumigatus*, fungal infections, innate immunity, adaptive immunity, cell therapy, cytokine therapy

## Abstract

Invasive aspergillosis (IA) is the most serious life-threatening infectious complication of intensive remission induction chemotherapy and allogeneic stem cell transplantation in patients with a variety of hematological malignancies. *Aspergillus fumigatus* is the most commonly isolated species from cases of IA. Despite the various improvements that have been made with preventative strategies and the development of antifungal drugs, there is an urgent need for new therapeutic approaches that focus on strategies to boost the host’s immune response, since immunological recovery is recognized as being the major determinant of the outcome of IA. Here, we aim to summarize current knowledge about a broad variety of immunotherapeutic approaches against IA, including therapies based on the transfer of distinct immune cell populations, and the administration of cytokines and antibodies.

## 1. Introduction

Within the last decade, the filamentous fungus *Aspergillus fumigatus* (*A. fumigatus*) has underlined its role as one of the most clinically relevant fungal pathogens. Conidia of this saprobic fungus can be isolated ubiquitously. Because of this high abundance, hundreds of spores of *Aspergillus* are inhaled daily by each individual [1]. Mucociliary clearance and phagocytic cells in the lung prevent disease in immunocompetent individuals. This includes alveolar macrophages, the major resident cells in the lung alveoli, which most efficiently engulf conidia in the lung [2]. 

The most severe disease caused by *Aspergillus* is invasive aspergillosis (IA). Major risk factors include immunosuppression, neutropenia, lymphopenia, and depletion of T cells [3]. Thus, IA occurs almost exclusively in immunocompromised patients. The incidence in allogeneic stem cell transplantation (allo-SCT) patients ranges from 4%–10%. Although many *Aspergilli* cause IA, *A. fumigatus* is responsible for more than 90% of all systemic *Aspergillus* infections [4]. 

Innate immunity is of major importance for the defense against *A. fumigatus*. In contrast to most bacterial pathogens, *A. fumigatus* undergoes major morphological changes during the early phase of infection. In the alveoli, inert spores swell, germinate, and grow into lung tissue, becoming subsequently angioinvasive and lastly undergoing hematogenous dissemination [5]. Cells of the innate immunity recognize the different fungal morphologies by distinct pattern recognition receptors (e.g., TLR2, TLR4, and dectin-1), which induce cell-specific and general defense mechanisms [6]. Upon stimulation with *A. fumigatus* in vitro, polymorphonuclear neutrophils (PMNs) release reactive oxygen intermediates and form neutrophil extracellular traps. Dendritic cells (DCs) release inflammatory cytokines (e.g., TNF-α and IL-1) and chemokines (e.g., IL-8 and CXCL10), which attract and activate other innate immune cell populations and build a bridge between the innate and the adaptive immunity by processing fungal antigens and presenting them to T cells. Natural killer (NK) cells degranulate and secrete cytotoxic proteins (perforin, granzymes) in response to *A. fumigatus*, causing fungal damage, and produce Th1 cytokines and chemokines, again attracting and activating other innate immune cell populations [7,8,9].

Unfortunately, there is still a lack of reliable diagnostic and therapeutic tools, resulting in high mortality rates of up to 90%, depending on the patient cohort and the localization of the fungus [10]. Therapy of *Aspergillus* infection remains limited to only a handful of antifungal agents. Voriconazole is the drug of choice for primary therapy of IA, with isavuconazole and the liposomal formulation of amphotericin B serving as alternatives. Echinocandins (e.g., anidulafungin) and other mold-active azoles (e.g., itraconazole and posaconazole) remain for salvage therapy [11]. Recently, triazole-resistant *A. fumigatus* strains have increasingly been isolated from patients. These strains emerge most likely due to the extensive use of azole fungicidals in agriculture and massively hinder antifungal treatment [12].

**Figure 1:**
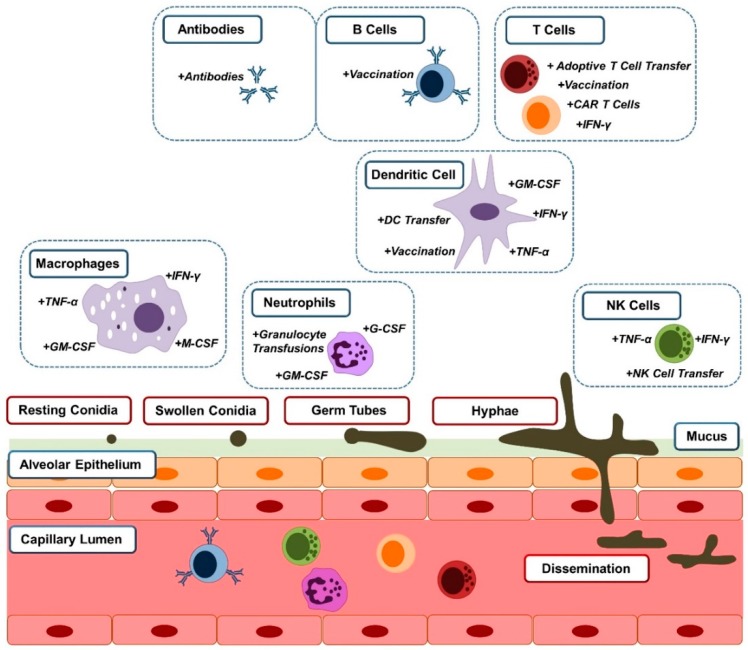
Cells of the innate and adaptive immune systems interact with different morphotypes of *Aspergillus fumigatus*. Macrophages clear resting and swollen conidia. Neutrophils attack all morphotypes of the fungus, while natural killer (NK) cells react to germ tubes and hyphae. Dendritic cells bridge the innate immune system to the adaptive immune system, which orchestrates fungal clearance of all morphotypes. Immunotherapeutic treatment options supporting these cells of the innate and adaptive immune systems are indicated next to the cell type they affect. Abbreviations: dendritic cell (DC); chimeric antigen receptor (CAR); granulocyte-macrophage colony stimulating factor (GM-CSF); granulocyte CSF (G-CSF); macrophage CSF (M-CSF); interferon (IFN) γ; tumor necrosis factor (TNF) α.

The following pages describe how different options of immune modulation have or will become alternatives to treat *Aspergillus* infection. In the case of immunocompromised patients, this usually involves therapeutic enhancement of immunity, including cell therapy approaches such as the transfusion of cells of the innate (granulocytes, dendritic cells, natural killer cells) and adaptive immune systems (T cells) as well as the administration of different cytokines, chemokines, and antibodies (Figure 1).

## 2. Cell Therapy

### 2.1. Granulocyte Transfusion

PMNs are able to engulf fungus, release antimicrobial peptides, and form extracellular traps [13,14]. After allo-SCT, especially during neutropenia, the ability of the immune system to effectively clear fungus is severely limited. Numerous studies have evaluated the transfusion of high numbers of neutrophils to patients during neutropenia in the past decades. Allogeneic granulocyte transfusions (GTs) dramatically increase neutrophil counts, which is speculated to reverse the increased susceptibility to infections in allo-SCT patients [15,16]. GTs have shown low toxicity in allo-SCT patients and are considered to be safe [16,17].

In a phase I/II clinical trial, allogeneic neutrophil transfusion in combination with dexamethasone and granulocyte colony stimulating factors (G-CSF) increased neutrophil counts and response; however, none of the five patients suffering from aspergillosis survived [16]. Mousset et al. reported that in hematological patients, who received either prophylactic or interventional GTs, *Aspergillus* infection could be controlled in 17 out of 22 cases. This result, however, was limited by the inclusion of possible IA cases into the study population and the trial’s nonrandomized nature [18]. In contrast, in a randomized phase III clinical trial in which GTs were given to neutropenic patients, no difference in 100-day survival of fungal infections was found. The authors of the study did not discriminate between different fungal infections; nonetheless, 49 of the 55 cases were *Aspergillus* infections [17]. In a randomized multicenter controlled study, 58 neutropenic subjects were treated with GTs plus G-CSF and dexamethasone in addition to standard microbial treatment. This arm of the trial was compared to 56 neutropenic patients on standard microbial treatment alone. No difference in infections between the groups was found. Both the control and treatment groups included only three proven aspergillosis cases [19], which made it difficult to draw conclusions for *Aspergillus* infections. Moreover, to improve the limited life span of transfused granulocytes, granulocyte progenitors for transfusion could be used. Bitmansour et al. have shown protection of neutropenic mice from *A. fumigatus* infection by granulocyte/monocyte progenitors [20,21].

In summary, although GTs have a lot of potential and new trials should be performed to further clarify the effect of GTs, no recommendation of treating allo-SCT patients with GTs is currently given [22]. The limited success of GT transfusions up to date might be a result of the transfusion of too low granulocyte numbers in some patients [19] and points to the necessity of overcoming multifactorial dysfunctions of the immune system after allo-SCT in order to prevent and clear IA. 

### 2.2. Dendritic Cells (DCs)

DCs connect innate and adaptive immunity. They recognize fungus by pattern recognition receptors and process fungal antigens. After activation, they secrete cytokines and chemokines and migrate to the lymph nodes. Here they present these antigens to specific T cells, which in turn are activated and primed. Ex vivo DCs stimulated with *Aspergillus* antigens induce protective immune responses to the fungus after transfusion to the patient due to activation of *Aspergillus*-specific T cells and secretion of cytokines and chemokines, which support the clearance of the fungus by both the innate and adaptive immune systems [23,24,25].

DC stimulation with unmethylated CpG oligodeoxynucleotides as an adjuvant in combination with one of the major *A. fumigatus* allergens, Asp f 16, induced a protective Th1 response in a hematopoietic stem cell transplantation (HSCT) mouse model of IA [26]. The same protective Th1 response was found when DCs were stimulated by *A. fumigatus* conidia and transfected with IL-12 in a similar murine model [27]. In addition, DCs that had been transduced with IL-12 and stimulated by *A. fumigatus* were administered to neutropenic mice in a model for IA. The treatment led to less mortality and decreased fungal burden due to a strong Th1 response [28]. Asp f 16-stimulated DCs were more effective in generating a cytotoxic T lymphocyte response against *Aspergillus* when antigen presentation of DCs was succeeded by a second antigen presentation using Epstein–Barr virus-transformed B lymphoblastoid cell lines. This method was described as more effective in generating Asp f 16-specific cytotoxic T lymphocytes (CTLs), and therefore would require less initial blood volume of the patient compared to DC stimulation alone [29].

In conclusion, an ex vivo stimulation of DCs and subsequent administration to the patient is cost inefficient, difficult to scale, and labor intensive. It shows, however, the therapeutic potential of fungal vaccination [30].

### 2.3. Natural Killer Cell Therapy

NK cells participate in the control of numerous pathogens, including viruses and fungi [31]. They have been shown to interact with *A. fumigatus, Cryptococcus neoformans, Candida albicans*, and Mucorales [32].

NK cells directly interact with *A. fumigatus* through the neural cell adhesion molecule (NCAM-1, CD56), and this interaction leads to the secretion of CC chemokine ligands CCL3, 4, and 5 [33]. After contact with *A. fumigatus*, NK cells become activated and release soluble factors such as perforin and granzyme, which mediate antifungal activity [34]. 

Higher reactive oxygen species (ROS) production and NK cell counts were associated with better control of IA in allo-SCT patients [35]. Referring to this study, Fernández-Ruiz et al. investigated NK cell counts of solid organ transplant recipients and correlated them to fungal infections. During the median follow-up period of 504.5 days, 10 out of 396 patients suffered from invasive fungal infection (IFI), and 4/10 IFI cases were classified as IA. Higher NK cell counts one month after transplantation decreased the incidence of fungal infections. In vivo, NK cells were the most significant contributor to IFN-γ secretion during the early stages of *Aspergillus* infection in the lungs of neutropenic mice. NK cell depletion resulted in higher mortality. In turn, fungal clearance was increased by transferring activated NK cells of wild-type mice to IFN-γ-deficient or wild-type neutropenic mice. The transfer of NK cells of IFN-γ-deficient mice into the same murine models, however, showed no effect [36]. Moreover, *Aspergillus niger* growth was partly inhibited due to increased NK cell activity in a murine model [37].

These results suggest that allogeneic NK cell transfer might be beneficial for the prevention of IA. Allogeneic NK cell transfer and transfer of the cell line NK92 after irradiation, which is already FDA-approved for clinical testing in certain types of cancer, were used in clinical studies and have a good safety profile in patients [38,39,40,41,42]. Nonetheless, they have to show their efficacy against IA in future studies.

### 2.4. Adoptive T Cell Transfer

The protective effect of *Aspergillus*-specific CD4^+^ cells of the Th1 lineage has been shown throughout the literature [43]. After allo-SCT, the adaptive immune system reconstitutes much slower than the innate immune system. Only a few *Aspergillus*-specific T cells can be measured 9–12 months after allo-SCT [44]. Therefore, an artificial increase of these specific T cells might help to clear *Aspergillus* in immunocompromised patients. For adoptive T cell transfer, T cells are isolated from the patient and stimulated with defined antigens. Consequently, T cell populations that are specific for the antigens are activated and proliferate. In turn, high numbers of these specific T cells are injected into the patient, where they recognize their target and aid the immune system in its elimination [45]. While the benefits of adoptive T cell transfer were illustrated in viral infections after transplantation, the development of similar techniques for the transfer of fungus-specific T cells lags behind [45]. 

One major obstacle to successful specific T cell transfer for *Aspergillus* in allo-SCT patients is the generation of an adequate number of *Aspergillus*-specific T cells with sufficient purity, using Good Manufacturing Practice (GMP) guidelines. Because of the fast progression of IA, the enrichment process needs to be as fast as possible. Many groups have worked toward complying with these requirements [46,47,48,49]. Bacher et al. reported a GMP-compliant protocol, in which they were able to enrich *Aspergillus*-specific T cells 200-fold in the T cell population. After isolation, they depleted cytotoxic and regulatory T cells, stimulated the remaining T cells with a GMP-grade *A. fumigatus* lysate, and isolated *A. fumigatus*-specific T cells with the help of the T cell activation marker CD137. This protocol is being used in an ongoing clinical trial (EudraCT Nr.2013-002914-11) [50]. However, another group has demonstrated that immunosuppressants such as cyclosporine A, methylprednisolone, as well as mycophenolic acid, lowered the number and activation of *Aspergillus*-specific protective Th1 cells. These immunosuppressants are frequently used after allo-SCT, complicating the application of adoptive T cell transfer in allo-SCT patients [51]. 

Up to this point, to our knowledge, there is only one clinical trial testing the efficacy and safety of adoptive T cell transfer in invasive fungal diseases. In this study, 10 patients with IA were treated by adoptive T cell transfer, while 13 IA patients in the control group did not receive a cell transfusion. The IA clearance rate was 90% in the treatment group compared to 53% in the control group. Infused cells did not cause graft versus host disease (GvHD) and showed a high IFN-γ to IL-10 ratio, indicating Th1 priming in the first three weeks after infusion. In contrast, patients in the control group had only a few naturally occurring *Aspergillus*-specific T cells 9–12 months after transplantation, exhibiting a nonprotective Th2 profile. In addition, patients receiving adoptive T cell transfer showed significantly decreased galactomannan antigenemia in comparison to the levels in the control group [44]. In order to shorten the time in between diagnosis and the transfusion of the cell product, off-the-shelf T cells specific for certain viruses were developed. They were found to be safe and only rarely caused mild GvHD (grade 1) [52]. Production of off-the-shelf T cells for transfusing IA patients is desirable. 

Current findings have indicated that not only CD4^+^ T cell responses are important against *Aspergillus*, but cytotoxic CD8^+^ T cells might play an important role as well [53,54]. CTLs stimulated with Asp f 16 were able to induce increased Th1 responses. The transfer of Asp f 16-specific CTLs resulted in higher survival in a murine model of IA [55]. In consequence, adoptive T cell transfer for IA might not only be limited to CD4^+^ cells. 

Adoptive T cell transfer is a promising tool for the fight against IA. GMP-compliant protocols for the production of sufficient numbers of *Aspergillus*-specific T cells are available, and off-the-shelf cell products against the infection might be developed soon. The results of the first clinical trial have been very promising, and there should be strong incentives for additional clinical trials. 

### 2.5. Chimeric Antigen Receptors (CARs)

**Figure 2:**
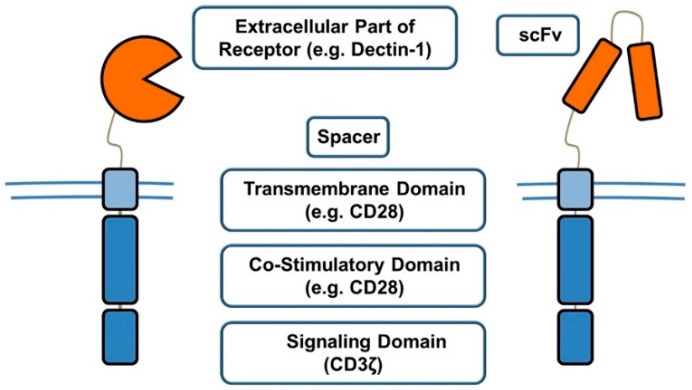
Second-generation chimeric antigen receptors (CARs) include a spacer, transmembrane domain, costimulatory domain, and signaling domain, as well as a targeting element. The two depicted CARs differ in their targeting element, which is usually the single chain variable fragment (scFv) of an antibody, but can also consist of the extracellular part of a receptor.

Chimeric antigen receptor (CAR) T cells are one of the most promising immunotherapeutic tools available and have shown their efficacy in primary clinical trials of B cell malignancies [56]. The FDA has already approved CAR T cell therapies for the treatment of acute lymphoblastic leukemia (ALL) and B cell lymphoma in appropriate patient groups [57]. CARs are artificially designed receptors that are introduced into T cells. MHC unrestricted antigen recognition and the capability to recognize glycoproteins and lipids are only two of the many advantages of this approach [58,59]. CARs consist of two extracellular, one transmembrane, and one intracellular element. The task of the extracellular targeting element is to recognize the target. The single chain variable fragment (scFv) region of a monoclonal antibody targeting the desired antigen is usually used, but other targeting elements such as extracellular parts of naturally occurring receptors are also tested. The targeting element is linked to a spacer or linker, which gives the targeting element the flexibility to bind to the intended target [60]. The most common transmembrane domain used in CARs is the transmembrane spanning region of CD28 [61]. The intracellular domain assures signal delivery, resulting in the activation of the cell. In first-generation CARs, CD3-ζ was used for signal transduction. In second-generation CARs, a costimulatory domain, most commonly CD28 and 4-1BB, was added to CD3-ζ, resulting in better persistence of CAR T cells [62] (Figure 2). 

The success of CAR T cells in B cell malignancies led to the attempt to use CARs for infections like aspergillosis. The group of Cooper et al. swapped the CD19 targeting element of a second-generation CAR, which is currently being evaluated in a clinical trial, with the extracellular part of Dectin-1 [63,64]. Dectin-1 is a naturally occurring receptor of the innate immune system that is not expressed on T cells. Its ligand ß-glucan is a polysaccharide found on the surface of many fungi, including *Aspergillus*. In addition to the extracellular part of Dectin-1, the Dectin-1 CAR (D-CAR) consisted of an IgG4 spacer, a CD28 transmembrane domain, and an intracellular domain of CD28 and CD3-ζ. The authors demonstrated that the D-CAR was activated by ß-glucan and inhibited the growth of *A. fumigatus*. In addition, IFN-γ concentration increased after stimulation, and the perforin/granzyme pathway was likely activated [63]. Interestingly, steroid treatment did not inhibit the antifungal activity of the D-CAR. 

In light of this report, CAR T cells might not only be helpful for the treatment of B cell malignancies, but also for *Aspergillus* infections. However, there is only one report about the efficacy of CAR T cells against *Aspergillus* available, and more data needs to be generated. Additional CAR constructs containing alternative costimulatory domains and new targeting elements could be more efficient and should be evaluated. Moreover, the autologous generation of sufficient numbers of CAR T cells takes from just over one up to several weeks, which might lose critical time in an acute infection like IA [60]. 

## 3. Cytokine Therapy

One approach to fight aspergillosis after allo-SCT is to strengthen the immune system by administering cytokines. The most discussed cytokines are available as recombinant forms approved by the FDA, resulting in a more efficient evaluation on new patient cohorts. Cytokine therapies aimed at innate or both innate and adaptive immune systems have been assessed in several studies.

### 3.1. Colony Stimulating Factors

CSF treatment is aimed at increasing the capacity of the innate immune system to clear *Aspergillus*. This might be achieved by a faster reconstitution of innate immune cells mitigating risk factors such as neutropenia as well as increasing the activity of these cells against the fungus. 

#### 3.1.1. G-CSF

G-CSF increases neutrophil proliferation as well as maturation and is FDA-approved [65]. After stem cell transplantation, it is frequently administered during febrile episodes of neutropenia. Even though G-CSF does not decrease mortality caused by infections, it reduces time of neutropenia and febrile neutropenia-related hospitalization periods [66]. In an *A. fumigatus* mouse model, the addition of G-CSF to the antifungal caspofungin or caspofungin combined with amphotericin B-intralipid, resulted in higher survival rates of up to 78.9%, decreased fungal burden in organs, and reduced serum galactomannan [67]. It also increased neutrophil counts and led to a four-fold higher killing of *A. fumigatus* conidia by PMNs compared to untreated controls [68]. 

Research shows that G-CSF shortens neutropenia in patients, but more studies investigating the effects of G-CSF on the prevalence and outcomes of *Aspergillus* infections have to be conducted [69,70]. 

#### 3.1.2. M-CSF

In contrast to G-CSF and granulocyte-macrophage CSF (GM-CSF), macrophage CSF (M-CSF) is not FDA-approved. Its main function is the stimulation of macrophage growth [71]. In a clinical phase I/II trial, the regular antifungal therapy of 46 bone marrow transplant patients was supplemented by recombinant human M-CSF. While the clinical outcome of patients infected with various *Candida* species improved compared to historical controls, no positive effect on patients suffering from aspergillosis was observed [72]. Treating transplanted mice with M-CSF before *A. fumigatus* challenge not only reduced fungal organ burden, but also increased survival rates from 10% in saline-treated animals to 60% [73]. Prophylactic administration of M-CSF to neutropenic rabbits in a model of pulmonary aspergillosis lowered pulmonary injury and increased survival, most likely due to increased macrophage numbers and phagocytosis activity [74]. 

Only a few experiments have been performed with M-CSF. M-CSF treatment has shown some promise in animal models for aspergillosis and should be evaluated further for the treatment of patients after allo-SCT.

#### 3.1.3. GM-CSF

Like G-CSF, GM-CSF is FDA-approved, but has a broader effect on immune cells. It plays a role in the differentiation of dendritic cells, as well as macrophages, and stimulates the proliferation and activation of many cell types, including neutrophils, macrophages, eosinophils, and dendritic cells [75,76]. Therefore, GM-CSF treatment increases numbers of tissue macrophages, circulating monocytes, neutrophils, and platelets, as well as eosinophils [77,78].

After allo-SCT, GM-CSF administration is considered safe [79]. In a prospective multicenter randomized phase IV clinical trial, 206 allogeneic stem cell patients were prophylactically administered with either G-CSF or GM-CSF alone, or a combination of both. Although GM-CSF and GM-CSF + G-CSF decreased combined 600-day IFI-related mortality and yeast incidence, no benefit for IA incidence was found [78].

GM-CSF might partly mitigate the effect of certain immunosuppressive drugs but inhibit the ability of the immune system to clear *Aspergillus*. Brummer et al. illustrated that GM-CSF prevents the immunosuppressive effects of dexamethasone on murine bronchoalveolar macrophages, leading to increased killing of *A. fumigatus* conidia [80]. Supportively, GM-CSF exposure lowered the fungal burden in the lung among cyclophosphamide immunosuppressed mice in a model of pulmonary aspergillosis [81]. Macrophage suppression by the corticosteroid cortisone acetate was also inhibited by GM-CSF. This effect lasted for more than a week after treatment in a murine model. In addition, GM-CSF has been shown to counteract corticosteroid-induced downregulation of pro-inflammatory cytokines such as TNF-α, which are crucial to early defense mechanisms of the innate immune system against *Aspergillus* conidia [82].

Even though GM-CSF shortens the time of neutropenia, which is the major risk factor for IA, no benefit concerning incidence or course of IA in larger patient cohorts has been found to date. Again, this demonstrates that addressing one of the many dysfunctions of the immune system after allo-SCT might not be sufficient to prevent or clear IA. However, GM-CSF potential to partly reverse undesired immunosuppressive effects after allo-SCT, which hamper infection control, might be an additional advantage of GM-CSF administration.

### 3.2. IFN-γ

A strong Th1 response is essential to clear *Aspergillus* [83,84,85]. In order to increase the Th1 response of patients, FDA-approved forms of IFN-γ might be administered. In vivo, IFN-γ is secreted by T and NK cells. It has the capacity to induce protective responses of the innate and adaptive immune systems against *Aspergillus* [43]. Numerous clinical studies have investigated the benefit of supplementing antifungal therapy with IFN-γ.

Case reports describing the positive effect of adjunctive IFN-γ administration on aspergillosis have been published [86,87,88,89]. In a randomized prospective placebo-controlled double-blinded clinical study of 128 patients undergoing chronic granulomatous disease, decreased frequencies of infections were observed after frequent IFN-γ administration compared to controls. However, only one patient in the IFN-γ-treated group and four patients in the placebo group suffered from aspergillosis, which highly limited the predictive value of the study. The study was also limited by the short follow-up period of only 10 months [90]. A case series of IFIs on renal transplant patients included three patients suffering from disseminated IA. All three cases were cured after six weeks of combined amphotericin B and IFN-γ treatment [91]. In a prospective case series of eight patients, including three aspergillosis cases, Delsing et al. showed increased ability of peripheral blood mononuclear cells (PBMCs) to produce pro-inflammatory cytokines IL-1β and TNF-α, Th17-stimulating cytokines IL-17 and IL-22, and heightened HLA-DR expression after combined IFN-γ and antifungal treatment, all of which play an important role in protecting the host from IA. While lymphocyte and monocyte numbers were increased, granulocyte numbers were slightly decreased [92]. In order to reverse the drop in granulocyte numbers, the addition of IFN-γ with a granulocyte count-increasing cytokine such as GM-CSF might be beneficial. Combination therapy of IFN-γ and GM-CSF, supporting antifungal treatment in two HIV-negative and one HIV-positive patient suffering from progressive pulmonary aspergillosis, showed promising results. Peripheral leukocyte numbers increased and Th1 response was strengthened. This resulted in an improved control of the fungal infection [93]. In vitro, pre-incubation of human PMNs with IFN-γ and GM-CSF led to enhanced *Aspergillus flavus* hyphal damage and increased release of oxygen radicals by PMNs. This effect disappeared when pre-incubating PMNs with only either one of the cytokines [94]. Another report demonstrated that IFN-γ treatment of PMNs and PBMCs resulted in increased hyphal damage of *A. fumigatus* [95]. 

Even though adjunctive IFN-γ treatment has many potential advantages and is well tolerated in allo-SCT patients [96], the evidence supporting the use in patients is still weak [22]. More trials need to be conducted. 

### 3.3. TNF-α

TNF-α is one of the most important cytokines in the defense against *Aspergillus* [97]. Comparable to IFN-γ, addition of TNF-α stimulates PMNs, which in turn increase oxygen radical release and cause enhanced hyphal damage against *A. fumigatus* in vitro. Although intracellular killing of *A. fumigatus* conidia by alveolar macrophages was not increased, phagocytosis was enhanced [98]. Administration of TNF-α to immunosuppressed mice in a model for pulmonary aspergillosis increased survival [99]. A time-dependent increase in TNF-α levels of the lung was correlated with higher migration of PMNs to the lung and increased survival of neutropenic and non-neutropenic mice after challenge with *A. fumigatus* conidia. In turn, blocking of TNF-α resulted in higher mortality and fungal lung burden in neutropenic mice. Prophylactic treatment of neutropenic mice with TNF-α increased their survival [100]. However, the major limitation of using TNF-α in the treatment of aspergillosis is its serious toxicity after systemic administration, including hepatotoxicity, nephrotoxicity, and neurotoxicity [101]. 

## 4. Other Immunotherapeutic Approaches

### 4.1. Vaccination

Successful vaccination elicits an adaptive immune response to a pathogen, leading to the generation of memory cells, which are able to fight subsequent infections with the same pathogen much more efficiently. There are different forms of vaccination available. First of all, inactivated whole-cell vaccines can be used; however, they have known limitations. They are complex and therefore difficult to standardize, and usually only elicit weak immune responses [102]. Live vaccines are more immunogenic, but are considered unsafe in immunocompromised patients, as they can potentially cause disease [103]. Subunit vaccines, which consist of purified elements, in combination with an adjuvant, could be the best method available in order to vaccinate immunocompromised patients, as they are easy to standardize and also considered safe in this cohort [53]. 

Subcutaneous vaccination using a hyphal sonicate protected immunocompromised mice in a model of IA [104]. The vaccination of mice with heat-killed *S. cerevisiae* before *A. fumigatus* challenge increased survival and decreased fungal organ burden. The major limitation of this study was the usage of immunocompetent mice without any immunosuppression. The usefulness of this approach in an immunocompromised setting cannot be predicted [105]. In another study, mice were vaccinated by intranasal inhalation of either filtrates of viable *A. fumigatus*, viable *A. fumigatus*, or heat-inactivated *A. fumigatus*. Thereafter, mice were immunosuppressed and challenged with the fungus. The filtrate and the live fungus vaccination were able to prolong survival and induced a protective Th1 response. In contrast, no prolonged protection was found in mice vaccinated with heat-inactivated fungus, which provoked a Th2 response [106]. Furthermore, vaccination with recombinant *Aspergillus* antigens Asp f 3, Asp f 9, Asp f 16 (all major allergens), Gel1 (a protein associated with cell wall morphogenesis), and Pep1 (an extracellular endopeptidase) resulted in protective effects in murine models of aspergillosis [107,108]. These antigens could potentially be used as the basis of subunit vaccines. The same is true for mannans, which can be found in the cell wall of *Aspergillus.* Liu et al. vaccinated immunocompetent mice with mannans derived from *C. albicans*, leading to increased survival rates after challenge with *A. fumigatus* conidia. Mortality was further decreased by the addition of bovine serum albumin (BSA) to the mannans [109].

Even though vaccine development in immunocompromised patients is difficult because of their weakened immune system, advances have been made to improve vaccination strategies in this patient cohort. However, T and B cell counts, as well as functionality, have to be at least partially restored in order to elicit a protective response. After allo-SCT, the reconstitution of the adaptive immune system takes several months [110]. Therefore, *Aspergillus* vaccination might not be effective in *Aspergillus* infections early after allo-SCT. It is still difficult and costly to develop vaccines against fungal pathogens, and in contrast to other microbes, no fungal vaccine has been licensed yet. Vaccines for other fungi, such as NDV-3A for *C. albicans*, which was used in a recent promising clinical trial, might lead to increased interest in the development of vaccines for *Aspergillus* [102,111]. 

### 4.2. Antibodies

Two decades ago, the humoral response was thought to play little to no role in the defense against fungi. More recent findings, however, show that this dogma needs to be revised. Humoral responses, in fact, are important for the host defense against fungal infection, including *Aspergillus* [112]. Patients with Good syndrome, a disease characterized by hypogammaglobulinemia, show increased incidences of fungal infections, including aspergillosis [113]. Anti-*Aspergillus* antibodies bind to swollen conidia and germ tubes, activating the classical pathway of the complement system. Complement activation leads to the killing of *Aspergillus* by neutrophils [114]. 

The efficacy of different kinds of antibodies against *Aspergillus* has been evaluated. One approach is to target polysaccharides found on the cell wall of fungi. The monoclonal antibody (mAb) 2G8 targets the cell wall polysaccharide laminarin, which consists of ß-glucan. It has demonstrated antifungal effects, including activity against *A. fumigatus* [115]. A different method is the usage of anti-idiotypic mAbs to yeast killer toxin, found in *Pichia anomala* and *Williopsis mrakii*, which displays antimicrobial effects. Administration of these mAbs to immunocompromised mice infected with *A. fumigatus* decreased fungal growth and increased survival [116]. Radioimmunotherapy has the potential to be another antibody-based immunotherapeutic strategy against *Aspergillus*. In this approach, an antibody directed against the fungus is tagged with radionuclides in order to deliver a lethal dose of radiation to the fungus [117]. 

The administration of antibodies might strengthen the ability of allo-SCT patients to prevent or clear IA in absence of a fully functional adaptive immune system. In contrast to adoptive T cell or CAR T cell transfer, in which autologous T cells have to be generated for each single patient over the period of weeks, one type of antibody would be instantly available for all affected patients, which might be a major advantage in acute infections. The research on the humoral response against *Aspergillus* and the implementation of immunotherapeutic strategies based on these findings is still in its early stages. However, the initial results generated are promising, and more data should be collected. 

## 5. Summary and Outlook

The treatment of IA patients with standard antifungal drugs faces numerous challenges. No new class of antifungal drugs has been invented for over a decade, the number of fungal isolates resistant to azoles has been steadily increasing, and the side effects of conventional antifungal drugs are still considered to be severe. Immunotherapeutic approaches hold promise for improving antifungal therapy in order to decrease high mortality rates. In general, immunotherapy protocols treating *Aspergillus* infections are still exploratory, cost-intensive, might be accompanied by severe side effects, and involve complex as well as time-intensive genetic and cellular manipulations before use. Different immunotherapeutic strategies have been investigated for their efficacy, safety, and their potential to overcome these challenges. The most promising candidates should be evaluated in well-designed clinical trials. Up to this point, the low prevalence of IA has first resulted in the clinical evaluation of these exploratory methods in small patient cohorts with low statistical power; and second, the analysis of a treatment’s efficacy has often been assessed in combined IFIs. Various IFIs are known to differ in their pathology and susceptibility to certain treatments. Thus, multicenter clinical trials for IA should be performed. 

There are promising weapons against *Aspergillus* on the horizon. In the future, this fight might involve the use of new NK CAR technology, a tool that can be used as an allogeneic “off-the-shelf” product [118]. It might also include checkpoint inhibitors. These molecules disable inhibiting receptors on immune cells and therefore increase their activity. They have demonstrated their efficacy in cancer research and might attenuate the clinical progression of IA [119]. Another promising approach is the usage of neutrophil-dendritic cell hybrids (PMN-DCs), which are cells with the microbicide function of PMNs and the capacity of DCs to stimulate adaptive immunity [120]. In addition, as drug development is expensive, and only a few drug candidates reach market maturity, the repurposing of approved drugs for potential use in IA might be worthwhile. Drugs such as auranofin and ebselen have shown activity against *Aspergillus* in vitro. Both drugs block the thioredoxin reductase pathway, which is essential for cells to manipulate disulfide bonds. This pathway is different in humans compared to bacteria, as well as fungi, and therefore might be a suitable target [121,122]. Moreover, combining novel immunotherapeutic approaches with antifungals might yield positive synergistic effects. For example, echinocandins are drugs that uncover immunologically active epitopes in the fungal cell wall. Many immunotherapeutic strategies such as D-CARs or ß-glucan-specific antibodies target these epitopes, which might result in more efficient fungal clearance [119].

In conclusion, the fight against IA still relies heavily on conventional antifungal drugs. Immunotherapy has made a lot of progress in the last decade and might be used as an adjuvant therapy or even on its own in the future. In order to bring these new treatment strategies to the bedside, well-designed multicenter clinical trials are of the upmost importance.
